# A Two-Phase Translational Framework for Personalized Ozone Therapy: From Baseline Clinical Redox Screening to Individualized *Ex Vivo* Concentration Selection

**DOI:** 10.3390/jpm16100510

**Published:** 2026-09-29

**Authors:** Ioannis Iliakis, Emmanouil Iliakis, Demitrios Vynios

**Affiliations:** 1 Ozonaim Clinic Athens, Efroniou 8, 11634 Athens, Greece; iiliakispmd@gmail.com (I.I.); driliakis.orth@gmail.com (E.I.); 2 Biochemistry, Biochemical Analysis and Matrix Pathobiochemistry Research Group, Department of Chemistry, University of Patras, 26501 Patras, Greece

**Keywords:** ozone therapy, oxidative stress, FRAS5, d-ROMs, PAT, personalized medicine, hormesis, musculoskeletal disorders

## Abstract

**Background**: Medical ozone therapy traditionally relies on standardized concentration protocols. However, interindividual variability in baseline oxidative stress and antioxidant capacity suggests that uniform dosing may be biologically suboptimal. **Objective**: To evaluate baseline redox heterogeneity in patients with musculoskeletal disorders and investigate whether an *ex vivo* graded ozone exposure framework using the FRAS5 system can identify personalized optimal redox response. **Methods**: In Phase 1 (Clinical Screening), baseline derivatives of reactive oxygen metabolites (d-ROMs), plasma antioxidant test (PAT), and the Oxidative Stress Index (OSI REDOX) were profiled in 11 symptomatic participants presenting with active pain. In Phase 2 (*Ex Vivo* Evaluation), whole blood samples from 9 consecutive participants were exposed to graded ozone concentrations (10–40 μg O_3_/mL O_2_) to map individual response curves. **Results**: Phase 1 revealed profound baseline heterogeneity despite identical clinical pain presentations, with OSI REDOX values varying nearly threefold (41–108), confirming highly divergent homeostatic starting points. Phase 2 *ex vivo* titration demonstrated non-linear, participant-specific redox trajectories. Multiple participants exhibited classic bi-phasic (hormetic) responses, achieving optimal OSI REDOX scores at intermediate concentrations (20–25 μg O_3_/mL O_2_), while higher concentrations triggered oxidative rebound or loss of antioxidant defense. **Conclusions**: Fixed ozone protocols fail to account for distinct individual redox profiles. Integrating baseline screening with *ex vivo* prospective profiling might offer a clinically accessible translational framework for biomarker-guided, personalized ozone concentration selection, after being confirmed in a larger cohort study.

## 1. Introduction

Medical ozone (O_3_) therapy exerts its therapeutic effects in musculoskeletal and inflammatory disorders by acting as a controlled chemical stimulus [1,2]. By generating transient, moderate oxidative stress, it triggers adaptive cellular responses, upregulating endogenous antioxidant enzymes and modulating inflammatory cascades [3,4]. Historically, the activity of medical ozone on intadiscal and pararvertebral structures has been grounded in clear histological and biochemical alterations [5].

Despite this dose-dependent biological mechanism, clinical practice frequently relies on fixed, semi-standardized concentration ranges applied uniformly across heterogeneous patient populations. This clinical approach assumes a uniform, linear dose–response relationship across all individuals. However, in disorders driven by tissue stress and chronic inflammation, patients enter treatment with fundamentally different levels of systemic oxidative strain and antioxidant reserve [6,7]. If an individual’s antioxidant defense is already near exhaustion, an empirical, mid-to-high dose of ozone may overwhelm their adaptive capacity, causing further oxidative imbalance rather than stimulation [1,4]. Recent extensive retrospective clinical evidence in 304 participants with peripheral neuropathy secondary to lumbar disorders demonstrated that clinical duration of effect is heavily influenced by concentration and age, revealing an optimal clinical target near 21 μg O_3_/mL O_2_ and a 1.8% decrease in efficacy duration for every unit increase above optimal levels [8]. Similarly, distinct clinical and demographic traits dictate outcomes in knee osteoarthritis treatments [9].

To bridge the gap between clinical outcomes and systemic metabolic status, biomarkers capable of characterizing real-time oxidative burden and antioxidant defense are required [10,11,12]. The Free Radical Analytical System (FRAS5:H&D s.r.l., Parma, Italy) provides an integrated clinic-accessible window into these parameters, utilizing the derivatives of reactive oxygen metabolites (d-ROMs) and the plasma antioxidant test (PAT) to evaluate the net Oxidative Stress Index (OSI REDOX) [11,13]. Elevated d-ROMs levels have been extensively validated as robust predictors of cardiovascular risk, systemic inflammatory disease activity, and outcomes in degenerative spinal disorders requiring surgical intervention [14,15,16,17].

To address this challenge, we developed a two-phase translational approach. First, we conducted a real-world clinical screening of baseline redox status in a series of 11 participants presenting with acute musculoskeletal complaints to characterize the extent of biological heterogeneity. Driven by the marked variation observed in this observational phase, we subsequently designed an *ex vivo* dose-titration protocol on a separate consecutive cohort of 9 participants. The overarching objective was to determine whether the FRAS5 system could track individual responses to graded ozone exposure, providing an empirical, mechanistic rationale for biomarker-guided, personalized ozone therapy based on the principles of metabolic hormesis [18,19].

## 2. Materials and Methods

### 2.1. Study Design and Ethical Considerations

The study followed a two-phase translational design, beginning with an observational clinical baseline screening of 11 adults with chronic musculoskeletal disorders to characterize real-world redox profiles using the FRAS5 platform, followed by an *ex vivo* graded-exposure experiment using blood samples from 9 consecutive participants to map personalized ozone-response concentration range; the full protocol received approval from the Ethical Committee of the University of Patras (16481/05-27-2024), and all participants provided written informed consent, with data pseudo-anonymized in accordance with international ethical standards and the Declaration of Helsinki.

### 2.2. Participant Cohorts and Sample Procurement

All participants across both phases presented with chronic musculoskeletal disorders (e.g., osteoarthritis, persistent low back pain, myofascial pain syndrome) accompanied by localized pain and functional impairment.

All 11 participants recruited for Phase 1 presented to the outpatient clinic with active, symptomatic pain and were evaluated immediately prior to receiving any clinical intervention. For one representative participant with severe oxidative stress, follow-up testing was performed one week after a standard medical ozone treatment session (21 μg O_3_/mL O_2_). A subsequent assessment, performed one week later, evaluated the combined effects of medical ozone therapy and multi-antioxidant supplementation (coenzyme Q10 and polyphenols).

For Phase 2, blood samples were collected using identical techniques and processed immediately to guarantee redox stability prior to gas exposure.

Ozone gas was synthesized from medical-grade oxygen using a certified medical ozone generator [Alnitec SRL, Cremosano, Italy]. Aliquots of whole blood were gently exposed to graded gas concentrations distributed across two exploratory mapping schemes, i.e., Protocol A (*n* = 6): 10, 25, and 40 μg O_3_/mL O_2_ and Protocol B (*n* = 3): 20, 25, and 30 μg O_3_/mL O_2_. Baseline values (0 μg O_3_/mL O_2_) served as the internal control for each participant.

### 2.3. Ex Vivo Redox Response Assessment

The *ex vivo* redox response assessment involved the preparation of four 5 mL syringes, leaving the first empty as a baseline control and filling the remaining three with ozone (2.5 mL) at predetermined concentrations; venous blood was then drawn into a 10 mL syringe and, using a 3-way stopcock, precisely 2.5 mL was distributed into each syringe before all mixtures were gently agitated for one minute to ensure uniform contact between blood and ozone.

### 2.4. Biomarker Quantification via FRAS5

Following agitation, a sample is drawn from each syringe individually. Redox monitoring was completed using the FRAS5 optical spectrophotometric system (H&D, Parma, Italy), using the following: 1. d-ROMs test, which measures plasma hydroperoxides via N,N-diethyl-p-phenylenediamine chromogen targeting; results are stated in Carratelli Units ($1\;U.\mathrm{Carr}=0.08\mathrm{mg}/\mathrm{dL}H_{2}O_{2}$) [10,12], 2. PAT, which evaluates the plasma non-enzymatic antioxidant barrier efficacy by tracking the reduction in ferric ions (Fe^3+^ to Fe^2+^) [11], and 3. OSI REDOX, which is a device-derived proprietary global index integrating absolute levels and the relative ratio of d-ROMs and PAT. Higher numbers signify severe shifts toward uncompensated oxidative stress.

### 2.5. Technical Procedure

During the first phase of the study, a blood sample may be obtained either by capillary finger-prick sampling, using a sterile lancet on the lateral side of a finger, according to the standard technique used for capillary blood glucose measurement, or by collecting a small amount of venous blood from a peripheral vein using a syringe.

Only a minimal amount of blood is required for analysis. Before blood collection, the sampling site is disinfected with 70% alcohol. Participants are advised to have the blood sample collected away from meals, in order to minimize the possible influence of recent food intake on the biochemical parameters under investigation.

The blood sample is transferred into the dedicated microvette and holder supplied as part of the FRAS5 test kit. The microvette is placed in the centrifuge incorporated into the FRAS5 system and centrifuged automatically according to the time and speed programmed in the instrument. The centrifugation separates the plasma from the cellular components of the blood. According to the current d-ROMs FAST instructions, centrifugation is performed for 90 s.

#### 2.5.1. d-ROMs FAST Test

The d-ROMs FAST test assesses the concentration of hydroperoxides (ROOH) in plasma and therefore provides an estimate of the patient’s pro-oxidant/oxidative status and is performed according to the supplier’s instructions.

#### 2.5.2. PAT

The PAT (Plasma Antioxidant Test) evaluates the antioxidant potential of plasma by measuring its ability to reduce ferric ions (Fe^3+^) to ferrous ions (Fe^2+^). The reaction is based on a ferric-ion source and a chromogenic system containing thiocyanate, with the resulting color change measured photometrically. The analysis was carried out according to the supplier’s instructions.

#### 2.5.3. Automated Calculation and Recording

The entire analytical sequence is electronically guided by the FRAS5 instrument. The device guides the operator through the successive steps of the procedure and automatically performs the photometric measurements. At completion of the analysis, the FRAS5 provides the d-ROMs and PAT values and calculates the Oxidative Stress Index (OSI). The results are automatically recorded and can be printed using the instrument’s integrated printer.

### 2.6. Statistical and Descriptive Analysis

Given the exploratory pilot design, statistical analysis was limited to descriptive parameters, ranges, and participant mapping trends to visualize response topologies (linear vs. bi-phasic). Missing data points (noted in one participant segment due to an automated sample volume error) were managed using pairwise deletion.

## 3. Results

The baseline screening data from 11 participants were first considered to characterize inter-individual differences in redox status before graded ozone exposure. Baseline d-ROMs values ranged from 216 to 427 U.Carr, PAT values from 1426 to 2045 U.Cor, and OSI REDOX values from 41 to 108, demonstrating substantial variability in the initial redox phenotype (Table 1).

For the Phase 2 *ex vivo* study, individual changes in d-ROMs, PAT, and OSI REDOX were examined at each tested ozone concentration relative to the corresponding 0 μg O_3_/mL O_2_ baseline. The complete individual profiles are now presented in Table 2. Because the exposure protocols were individualized and included different concentration ranges (0–10–25–40 μg O_3_/mL O_2_ in six participants and 0–20–25–30 μg O_3_/mL O_2_ in three participants), we considered within-participant response patterns more informative than applying a single pooled dose–response model to all participants.

We also introduced an explicit exploratory criterion for the description of nonlinear or biphasic-like responses. A response was considered biphasic-like when a biomarker showed a directional change at lower ozone concentrations, followed by a reversal at an intermediate concentration and a subsequent change in the opposite direction at the highest tested concentration. When this pattern was not clearly present, the response was described as relatively flat, monotonic, or otherwise nonlinear according to the observed individual profile.

Using this approach, several participants demonstrated nonlinear or biphasic-like patterns, particularly for OSI REDOX. For example, CDPOR001 showed OSI REDOX values of 42, 68, 49, and 55 at 0, 10, 25, and 40 μg O_3_/mL O_2_, respectively. CDPOR0006 showed a more pronounced pattern, with values of 51, 38, 5, and 59 at the same concentrations. CDPOR0007 demonstrated a reduction from 77 at baseline to 26 and 28 at 20 and 25 μg O_3_/mL O_2_, respectively, followed by an increase to 45 at 30 μg O_3_/mL O_2_. In contrast, other participants showed relatively flat or different response patterns, such as CDPOR0003, whose OSI REDOX values remained within a relatively narrow range (11–15).

### 3.1. Phase 1: Baseline Clinical Screening Heterogeneity

Descriptive profiling of the Phase 1 cohort unveiled a striking degree of biological diversity. Crucially, although all 11 participants entered the clinic presenting with active clinical pain discomfort, their baseline systemic metabolic status and initial redox settings were profoundly different (Table 1).

**Figure 1:**
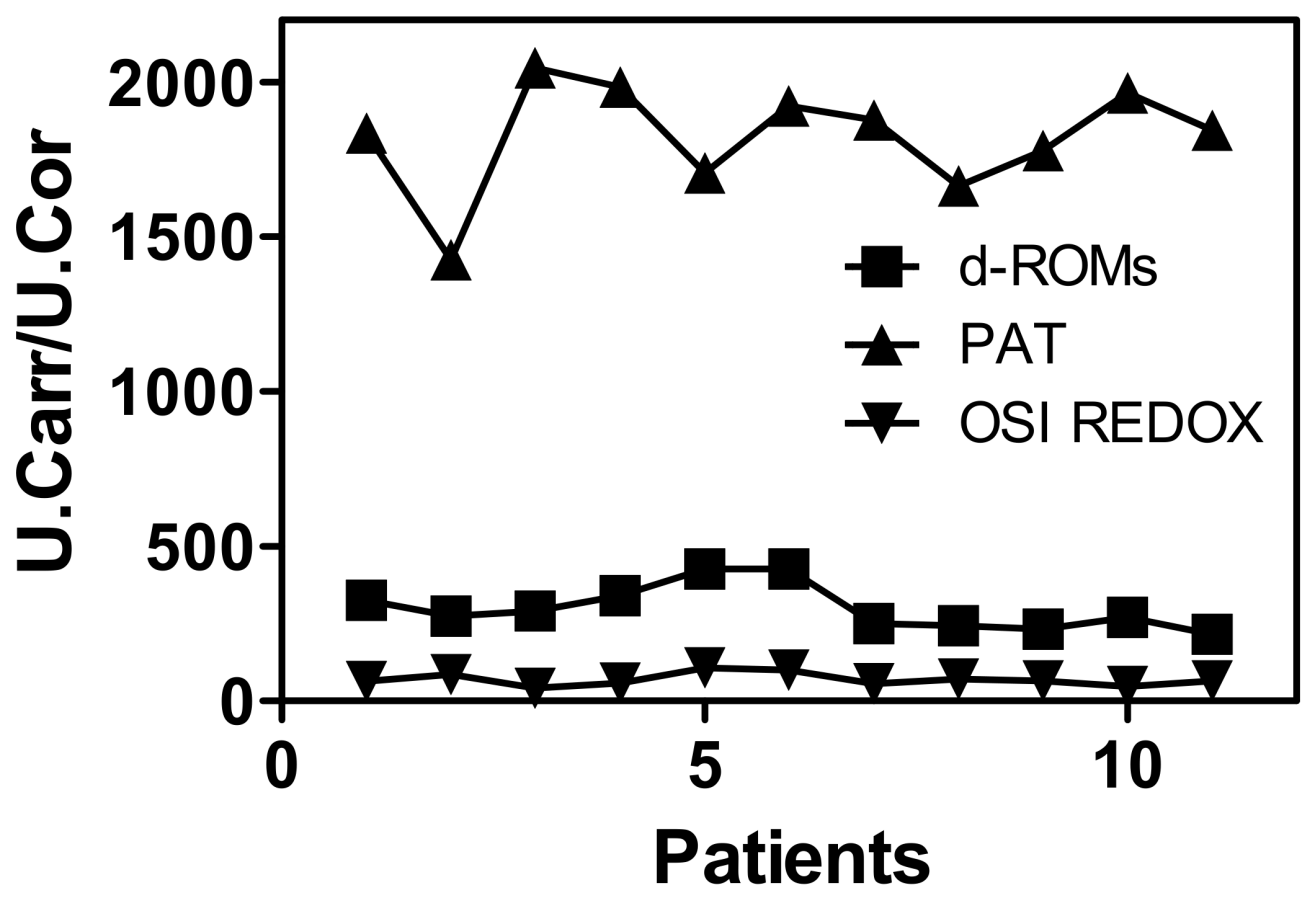
Individualized ozone concentration selection based on FRAS5 redox profiling: Evaluation of eleven cases using the FRAS5 system demonstrated considerable inter-participant variability in redox responses to different ozone concentrations. The absence of a uniform dose-dependent pattern supports the concept of personalized ozone concentration selection based on participant redox adaptation. For simplification, Arabic numbering of participants is used.

Baseline hydroperoxide levels (d-ROMs) demonstrated a vast range, stretching from a low of 216 U.Carr (SCREEN11), indicating low baseline systemic oxidation, to a severely elevated peak of 427 U.Carr (SCREEN06) and 426 U.Carr (SCREEN05), denoting massive oxidative burden. Similarly, individual antioxidant buffer efficiency (PAT) varied considerably, with values ranging from a depleted state of 1426 U.Cor (SCREEN02) to a robust capacity of 2045 U.Cor (SCREEN03). Reflected against these structural imbalances, the global baseline OSI REDOX index values ranged from 41 to 108, nearly a threefold difference across the screening cohort. Figure 1 presents the profound systemic heterogeneity at baseline. This emphasizes that participants presenting with active musculoskeletal pain occupy entirely disparate metabolic starting points. The longitudinal subset tracks the sequential redox changes in participant SCREEN05, showing a reduction in oxidative burden following ozone administration, followed by a further improvement in the measured redox profile after antioxidant supplementation.

### 3.2. Treatment Case Snapshot (SCREEN05)

Within this screening cohort, participant SCREEN05 presented with a highly adverse initial redox profile (d-ROMs: 426, PAT: 1705, OSI: 108). Following a standard clinical ozone administration session (Post-ozone (21 μg O_3_/mL O_2_) treatment), the global index decreased to 76 (d-ROMs dropped to 360, PAT rose to 1798). Crucially, the subsequent addition of targeted oral antioxidants (Post-antioxidant supplementation: CoQ10 + Polyphenols) induced a further improvement in the measured redox profile, shifting the OSI down to 64 and increasing PAT to 1907.

### 3.3. Phase 2: Individualized Responses to Graded Ozone Exposure

**Figure 2:**
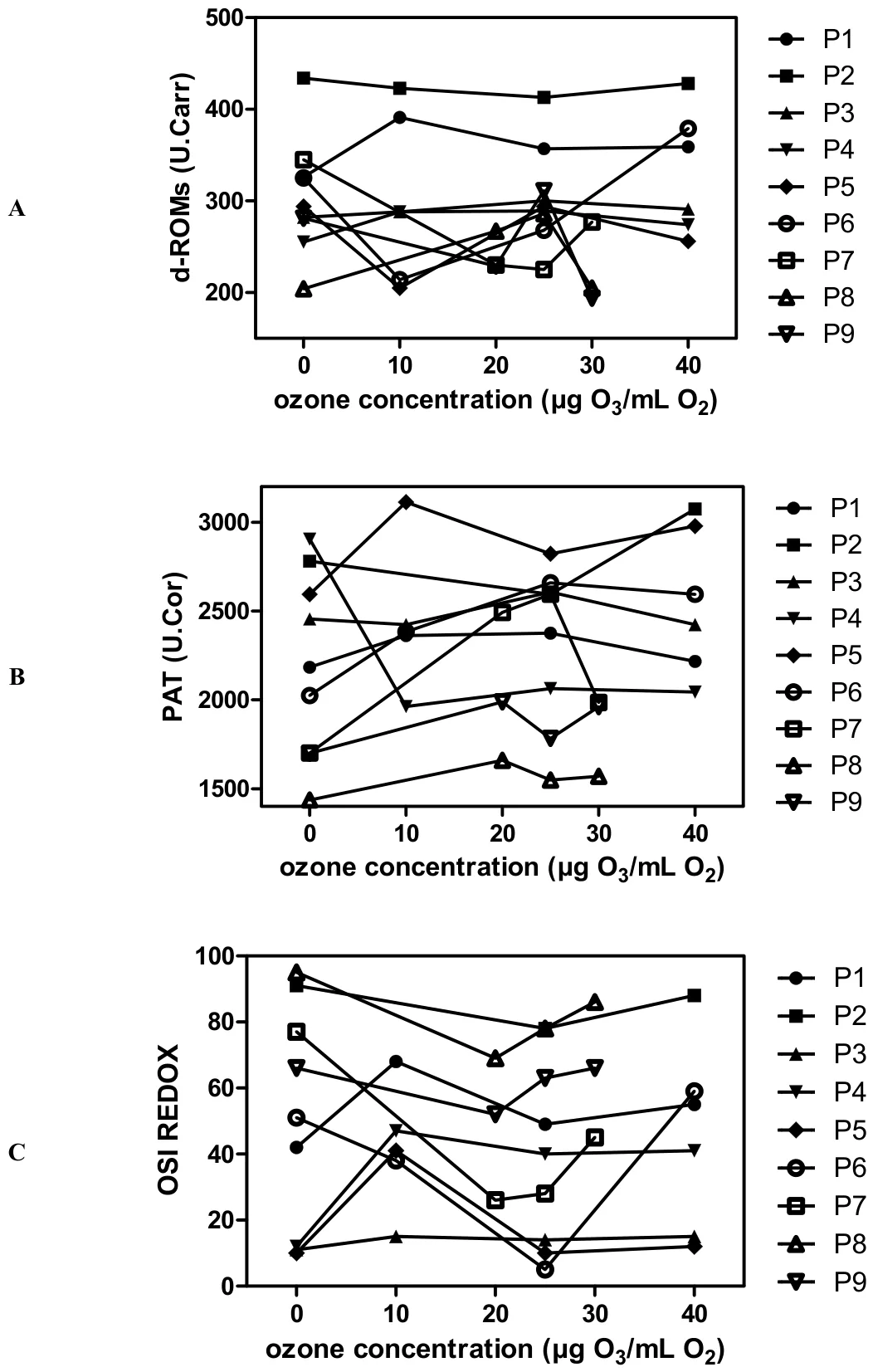
The analysis of nine participants revealed heterogeneous redox responses following exposure to different ozone concentrations. No consistent dose-dependent trend was observed across d-ROMs (**A**), PAT (**B**) and OSI Redox (**C**) parameters. Instead, everyone demonstrated a distinct redox adaptation pattern, highlighting the potential importance of personalized ozone concentration selection based on baseline oxidative/antioxidant status. For simplification, numbering of participants from P1 to P9 is used.

The experimental phase confirmed that the observed baseline redox diversity translated into highly heterogeneous, non-linear biological responses following *ex vivo* ozone exposure. No uniform or predictable dose–response relationship was identified across the nine participants (Table 2). As illustrated in Figure 2, graded ozone challenges elicited distinct participant-specific metabolic response patterns. Several participants exhibited nonlinear patterns, including patterns compatible with biphasic-like responses, consistent with the adaptive redox responses described in advanced autohemotherapy paradigms [1].

**Table 2:** Phase 2 Participant *Ex Vivo* Graded Ozone Profiles (n = 9).

Participant	Concentration (μg O_3_/mL O_2_)	d-ROMs (U.Carr)	PAT (U.Cor)	OSI REDOX
CDPOR001	0	326	2184	42
	10	391	2362	68
	25	357	2375	49
	40	359	2217	55
CDPOR0002	0	434	2780	91
	10	423	Missing ^	Missing ^
	25	413	2594	78
	40	428	3074	88
CDPOR0003	0	282	2455	11
	10	288	2423	15
	25	300	2607	14
	40	291	2423	15
CDPOR0004	0	255	2905	12
	10	288	1963	47
	25	289	2064	40
	40	274	2044	41
CDPOR0005	0	294	2594	10
	10	205	3113	41
	25	294	2822	10
	40	256	2979	12
CDPOR0006	0	325	2025	51
	10	214	2383	38
	25	268	2659	5
	40	379	2594	59
CDPOR0007	0	345	1700	77
	20	230	2492	26
	25	225	2594	28
	30	277	1986	45
CDPOR0008	0	204	1436	95
	20	267	1660	69
	25	286	1549	78
	30	205	1570	86
CDPOR0009	0	281	1699	66
	20	229	1989	52
	25	311	1783	63
	30	194	1962	66

^_Sampling handle limitation.

For example, participant CDPOR0006 showed a marked improvement in redox status, with the OSI REDOX index decreasing from 51 at baseline to 38 following exposure to 10 μg O_3_/mL O_2_, reaching a lowest observed *ex vivo* redox response value of 5 at 25 μg O_3_/mL O_2_ due to a reduction in circulating hydroperoxides and a concomitant increase in PAT to 2659, before rebounding to 59 at 40 μg O_3_/mL O_2_, indicating a reversal of the favorable redox response. Similarly, CDPOR0007 (Protocol B) demonstrated a favorable *ex vivo* redox response over the 20–25 μg O_3_/mL O_2_ range, where the OSI REDOX index declined from 77 to 26 and 28, but increased again to 45 following exposure to 30 μg O_3_/mL O_2_. In contrast, participant CDPOR0003 displayed a relatively unresponsive phenotype, with the OSI REDOX index remaining stable within a narrow range (11–15) across all ozone concentrations tested (10–40 μg O_3_/mL O_2_). Conversely, participant CDPOR0004 exhibited a hypersensitive response, with the lowest ozone concentration (10 μg O_3_/mL O_2_) causing a marked depletion of antioxidant capacity (PAT decreasing from 2905 to 1963) accompanied by an increase in the OSI REDOX index from 12 to 47, without evidence of recovery at higher ozone concentrations. Collectively, these findings demonstrate pronounced inter-participant variability in systemic redox responses and support the existence of an individualized concentration response range rather than a universal optimal ozone dose.

## 4. Discussion

The clinical and experimental data gathered across this two-phase project clarify a foundational aspect of redox medicine: the same dose of medical ozone does not equal the same biological response in all participants.

The widespread baseline values logged during Phase 1 demonstrate that participants with similar clinical pain presentations possess entirely distinct homeostatic settings. A participant starting with an OSI REDOX of 108 (such as SCREEN05) is in a substantially different oxidative state from one with an OSI REDOX of 41 (such as SCREEN03). This indicates that, although subjective pain measures remain clinically important, they do not necessarily reflect the underlying biomolecular stress environment.

When these distinct biological states are subjected to graded ozone exposure in Phase 2, the responses showed patterns compatible with a nonlinear or hormetic-like response to oxidative stimuli [4,18,19]. This was particularly evident in participants such as CDPOR0006 and CDPOR0007, whose responses were more favorable at intermediate concentrations (20–25 μg O_3_/mL O_2_), whereas higher concentrations were associated with acute decompensation. These findings are consistent with recent multi-center clinical observations, identifying approximately 21 μg O_3_/mL O_2_ as a concentration associated with peak efficiency in peripheral neuropathy, while higher concentrations were associated with reduced longevity of the clinical effect [8]. Conversely, the hypersensitive profiles (CDPOR0004) indicate that some participants may require highly cautious, ultra-low initial titration to avoid depleting vulnerable antioxidant reserves. Blood antioxidant levels are determined not only by antioxidant intake, including vitamins and polyphenols, but also by endogenous antioxidant capacity, particularly glutathione, superoxide dismutase, catalase, and glutathione peroxidase, as well as by oxidative challenges related to lifestyle, age, smoking, air pollution, and other environmental factors [20,21,22,23]. Recent literature further supports the biological plausibility of these findings, showing that controlled ozone-induced oxidative inputs may modulate dysregulated redox networks, attenuate NFκB-driven inflammation, and enhance mitochondrial resilience in musculoskeletal disorders [2,24].

Within the proposed framework, an initial clinical assessment would be followed by baseline redox profiling and *ex vivo* exposure of the participant’s blood to a limited range of ozone concentrations. The resulting individual response profile could then be used to identify a candidate concentration or concentration range for subsequent clinical treatment. Importantly, this approach remains hypothesis-generating and requires prospective clinical validation before it can be considered a dosing algorithm. It should not be interpreted as suggesting that a single biomarker or a single *ex vivo* measurement can independently determine the clinically optimal ozone concentration. Rather, d-ROMs, PAT, and OSI REDOX should be regarded as complementary indicators of individual redox response that may be integrated with clinical characteristics, treatment indication, and established clinical experience to support individualized dose selection.

Looking forward, the transition from *ex vivo* redox profiling to prospective, patient-specific dosage selection represents an important translational opportunity for ozone-based interventions. The integration of baseline oxidative-stress screening with individualized response curves could support a precision-redox medicine model in which ozone concentration is progressively matched to each participant’s biochemical resilience and adaptive capacity. Potential future developments include standardization of redox phenotypes, with clinically validated categories such as high-burden, balanced, and hypersensitive profiles; predictive modelling using machine-learning approaches trained on *ex vivo* response curves to estimate the safest and most effective concentration range; dynamic dose adjustment according to measurable changes in OSI REDOX, PAT, or d-ROMs; and clinical decision-support tools integrated into point-of-care devices for real-time selection of concentration ranges. Prospective trials should also determine whether biomarker-guided dosing improves the durability of analgesic effects, reduces rebound pain, and minimizes oxidative overload.

The long-term clinical impact of this approach could be a transition from fixed-dose protocols toward biomarker-guided personalization, potentially reducing treatment variability and enhancing therapeutic precision across heterogeneous inflammatory and neuropathic conditions. In this framework, *ex vivo* profiling would become not merely an experimental tool but a clinical compass for directing ozone concentration according to each participant’s unique redox physiology.

### Limitations

This study represents a real-world, hypothesis-generating pilot series. Its findings are bound by a small cumulative sample size and an *ex vivo* design that isolates vascular tissue from systemic neuroendocrine and endothelial counter-regulatory loops. Additionally, certain data limitations, such as a minor missing values occurrence in one participant step (CDPOR0002 at 10 μg O_3_/mL O_2_, require careful scaling. Finally, because the OSI REDOX is a device-derived proprietary score, clinicians must interpret its values alongside raw d-ROMs and PAT figures to maintain optimal clinical oversight.

## 5. Conclusions

Systemic redox profiles are highly individualized and cannot be predicted by clinical symptoms alone. Utilizing baseline screening to assess oxidative stress levels, combined with targeted *ex vivo* testing, seems to provide a reliable, clinically practical method to avoid standard, unmodulated dosing errors. This framework might establish a scientific foundation for personalized, biomarker-guided adjustments in medical ozone therapy.

### Future Clinical Development and Clinical Translation

A potential future development of the present approach is the transition from *ex vivo* redox profiling to prospective, individualized ozone dose selection. A stepwise model could include clinical assessment, characterization of baseline redox status, and *ex vivo* exposure of a blood sample to selected ozone concentrations. The resulting d-ROMs, PAT, and OSI REDOX profiles could then help identify a candidate concentration or concentration range for subsequent treatment. This concept is consistent with the proposed role of low-dose ozone as a controlled redox stimulus capable of inducing adaptive antioxidant responses [25,26].

The present findings may also be compatible with a nonlinear or hormetic-like response to oxidative stimuli. However, this study does not establish a hormetic dose–response relationship. The individual *ex vivo* profiles instead suggest that redox responses may vary between individuals and ozone concentrations. Therefore, these findings should be considered hypothesis-generating and should not yet be used as a validated dosing algorithm.

d-ROMs, PAT, and OSI REDOX provide complementary information on oxidative burden and antioxidant capacity, but they should not be regarded as direct surrogates of clinical outcome. In particular, d-ROM measurements require careful interpretation and standardization [10]. Future studies should therefore determine whether combinations of redox biomarkers can provide reproducible predictive information.

The clinical relevance of this approach is supported by the growing literature on ozone therapy in musculoskeletal disorders, including evidence concerning redox modulation, inflammation, and pain pathways [2,27]. A recent prospective randomized clinical study in patients with chronic musculoskeletal pain and abnormal mitochondrial redox status also reported changes in pain outcomes together with redox-related parameters following ozone treatment [28]. These findings provide a rationale for investigating whether baseline redox status and *ex vivo* response profiles can predict treatment response.

The next step should therefore be a prospective clinical validation study in which the *ex vivo* response is obtained before treatment and used to select a predefined ozone concentration or concentration range. This approach could be compared with a conventional fixed-concentration protocol using predefined clinical outcomes, safety parameters, and repeated redox measurements.

Thus, the principal clinical implication is not that FRAS5 profiling can already define a specific ozone concentration, but that individual redox-response profiling may provide a rational framework for future personalization. Larger, adequately powered studies with standardized protocols, predefined response criteria, repeated measurements, and clinically meaningful outcomes are required before translation into routine clinical practice.

## Figures and Tables

**Table 1:** Phase 1 Clinical Baseline Screening Profiles (n = 11).

Participant Code	Condition/Intervention Step	d-ROMs (U.Carr)	PAT (U.Cor)	OSI REDOX
SCREEN01	Baseline (0 μg O_3_)	326	1835	63
SCREEN02	Baseline (0 μg O_3_)	274	1426	86
SCREEN03	Baseline (0 μg O_3_)	290	2045	41
SCREEN04	Baseline (0 μg O_3_)	340	1982	58
SCREEN05	Baseline (0 μg O_3_)	426	1705	108
	Post-ozone (21 μg O_3_/mL O_2_) treatment	360	1798	76
	Post-antioxidant supplementation	342	1907	64
SCREEN06	Baseline (0 μg O_3_)	427	1922	100
SCREEN07	Baseline (0 μg O_3_)	250	1876	55
SCREEN08	Baseline (0 μg O_3_)	243	1664	71
SCREEN09	Baseline (0 μg O_3_)	232	1777	65
SCREEN10	Baseline (0 μg O_3_)	271	1965	47
SCREEN11	Baseline (0 μg O_3_)	216	1844	65

Abbreviation: d-ROMs: Derivatives of reactive oxygen metabolites; PAT: Plasma antioxidant test; OSI REDOX: Oxidative Stress Index.

## Data Availability

The original contributions presented in this study are included in the article. Further inquiries can be directed to the corresponding author.

## Abbreviations

The following abbreviations are used in this manuscript:

d-ROMs	Derivatives of reactive oxygen metabolites
PAT	Plasma antioxidant test
OSI REDOX	Oxidative Stress Index
FRAS5	Free Radical Analytical System
